# Cardio-kidney-metabolic complexity in patients with atrial fibrillation: an analysis from the prospective GLORIA-AF registry phase III

**DOI:** 10.1186/s12933-025-02950-y

**Published:** 2025-10-11

**Authors:** Giulio Francesco Romiti, Davide Antonio Mei, Bernadette Corica, Marco Proietti, Giuseppe Boriani, Brian Olshansky, Tze-Fan Chao, Menno V. Huisman, Gregory Y. H. Lip

**Affiliations:** 1 Liverpool Centre for Cardiovascular Science at University of Liverpool, Liverpool John Moores University and Liverpool Heart & Chest Hospital, Liverpool, UK; 2 Department of Translational and Precision Medicine, Sapienza – University of Rome, Rome, Italy; 3 Cardiology Division, Department of Biomedical, Metabolic and Neural Sciences, University of Modena and Reggio Emilia, Policlinico di Modena, Modena, Italy; 4 Department of Clinical Sciences and Community Health, University of Milan, Milan, Italy; 5 Division of Cardiogeriatric Subacute Care, IRCCS Istituti Clinici Scientifici Maugeri, Milan, Italy; 6 Division of Cardiology, Department of Medicine, University of Iowa, Iowa City, USA; 7 Division of Cardiology, Department of Medicine, Taipei Veterans General Hospital, Taipei, Taiwan; 8 Institute of Clinical Medicine, and Cardiovascular Research Center, National Yang Ming Chiao Tung University, Taipei, Taiwan; 9 Department of Thrombosis and Hemostasis, Leiden University Medical Center, Leiden, The Netherlands; 10 Department of Clinical Medicine, Aalborg University, Aalborg, Denmark; 11 Medical University of Bialystok, Bialystok, Poland

**Keywords:** Cardio-kidney-metabolic syndrome, Cardio-renal-metabolic syndrome, Atrial fibrillation, Clinical complexity

## Abstract

**Background:**

Cardio-Kidney-Metabolic (CKM) syndrome results from the complex interaction of cardiovascular, renal and metabolic comorbidities. Data on the epidemiology and clinical impact of the CKM syndrome in patients with atrial fibrillation (AF) are limited. We evaluated CKM domains and their impact in a real-world cohort of patients with AF.

**Methods:**

From the prospective global GLORIA-AF Registry phase III study, we defined CKM domains according to cardiovascular, renal and metabolic comorbidities or conditions in patients with AF and CHA_2_DS_2_-VASc score ≥ 1. We analysed the association of the number and groups of CKM domains with use of oral anticoagulant (OAC) and the risk of major outcomes via multiple-adjusted regression analyses. Our primary outcome was a composite of all-cause death and major adverse cardiovascular events.

**Results:**

16,070 patients (age 70.1 ± 10.4 years, 45.2% females) were included; 1931 (12.0%) presented with all 3 CKM domains, with substantial geographical variation in the distribution of CKM domains. OAC use increased with the number of CKM domains (Odds Ratio [OR] and 95% Confidence Intervals [CI]: 1.40 [1.14–1.72] and 1.38 [1.07–1.78] for 2 vs. 0 and 3 vs. 0 CKM domains, respectively). Over a 3-year follow-up, the incidence of the primary composite outcome increased with the number of CKM domains, with highest hazard observed in patients with 3 domains (Hazard Ratio [HR] and 95%CI: 1.69 [1.20–2.37]). Among groups of CKM domains, those characterized by the kidney domain showed the highest association with the risk of clinical outcomes.

**Conclusions:**

In patients with AF, CKM domains are commonly found, and their prevalence is heterogeneous across geographical regions. CKM syndrome influences OAC use and had detrimental prognostic effects, with an increasing risk of all-cause death and MACE as the burden of CKM domains increased.

**Supplementary Information:**

The online version contains supplementary material available at 10.1186/s12933-025-02950-y.

## Introduction

The Cardio-Kidney-Metabolic (CKM) syndrome, also referred to as cardio-renal-metabolic syndrome, is the result of the complex interaction of metabolic risk factors, chronic kidney disease, and cardiovascular diseases. The CKM syndrome predisposes to end-organ damage, and is responsible for a significant proportion of cardiovascular mortality worldwide [1, 2]. Importantly, CKM syndrome is progressive in nature, and can be classified into five stages, from absence of CKM risk factors (stage 0) to clinically-evident cardiovascular disease, occurring in the context of other CKM conditions (stage 4) [2].

Atrial fibrillation (AF), one of the late-stage clinical manifestations of the CKM syndrome [2], predisposes to mortality and the occurrence of other cardiovascular diseases, including stroke, thromboembolism and heart failure [3]. Historically, the burden of cardiovascular risk factors has been directly associated with the risk of adverse outcomes in patients with AF [4, 5]. More recently, the contribution of multimorbidity and “clinical complexity” on the trajectories of patients with AF has been acknowledged and reported [6-9]. For these reasons, increasing attention has been posed to the integrated and holistic management of patients with AF, in order to improve their prognosis [10].

Although AF is usually considered at the end of the CKM spectrum, whether the trajectories of patients with established AF are influenced by CKM syndrome (and therefore, CKM components) is not known.

In this study, we analyzed the association of CKM domains with the management and clinical outcomes of a contemporary cohort of patients with AF enrolled in the GLORIA-AF Registry Phase III.

## Materials and methods

We analyzed data from the GLORIA-AF Registry, an international, prospective, multicentre registry programme structured in 3 phases, designed to assess the real-world long-term efficacy and safety of dabigatran etexilate in patients with a recent diagnosed AF. Full details on the study design, study procedures and primary results of GLORIA-AF registry were already reported elsewhere [11–14]. For this analysis, we considered only the phase III of the registry, which enrolled adult patients (age ≥ 18) with a recent diagnosis of non-valvular AF (i.e. within 3 months, or within 4.5 months in Latin America), and a CHA_2_DS_2_-VASc score ≥ 1 between 2014 and 2016. Main exclusion criteria were AF due to a reversible cause, presence of a mechanical heart valve (or patients expected to undergo valve replacement), previous treatment with vitamin K antagonist (VKA) for > 60 days during their lifetime, other clinical indications for OAC, or a limited life expectancy (< 1 year). Approval of the study protocol was provided by local institutional review boards at each participating centre, and written informed consent was obtained from all patients. The study was conducted according to the Declaration of Helsinki and the Good Clinical Practice.

For the purposes of this analysis, we included only patients with complete data on i) the conditions defining each CKM domain and ii) the primary composite outcome (see below).

### Definition of the CKM syndrome and its component

To define components of the CKM syndrome, we considered data on clinical characteristics and comorbidities as collected at baseline visit by the study investigators, according to the definition used in the standardized case report forms. We considered the following “domains” of CKM syndrome, along with their qualifying criteria:

- Cardiovascular domain: presence of coronary artery disease (CAD), congestive heart failure (CHF; irrespective of ejection fraction), peripheral artery disease (PAD), or a history of stroke/transient ischemic attack (TIA);
- Kidney domain: a Creatinine Clearance (CrCl) < 60 ml/min, as calculated by the Cockroft-Gault formula;
- Metabolic domain: a body mass index (BMI) ≥ 25 kg/m^2^ (i.e., overweight or obesity status), diabetes mellitus, or hyperlipidemia.

Any patient presenting with at least one of the conditions or diseases listed above was considered as having that domain (i.e., component) of the CKM syndrome.

For subsequent analyses, we also considered the following variables:

- Numbers of CKM domains: either 0, 1, 2, or 3 domains
- Groups of CKM: patients with none (0) domains of the CKM syndrome; patients with only cardiovascular, kidney, or metabolic domain; patients with cardiovascular and kidney (cardio-kidney), cardiovascular and metabolic (cardio-metabolic) or kidney and metabolic (kidney-metabolic) domains; patients with all domains (cardio-kidney metabolic).

To analyze the associations of the number of CKM domains and CKM groups with the management of patients with AF, we considered treatments received at baseline. We considered antithrombotic use (i.e. use of OAC and type of OAC, either a VKA or a non-vitamin K antagonist oral anticoagulant [NOAC]), other drugs (i.e., angiotensin converting enzyme [ACE] inhibitors, angiotensin receptor blockers [ARB], diuretics, beta-blockers [either selective or non-selective], digoxin, verapamil/diltiazem, propafenone, flecainide, amiodarone, dronedarone, other antiarrhythmic drugs, statins, insulin and oral hypoglycaemic agents) and interventional procedures (AF ablation and cardioversion), as collected at baseline.

### Follow-up and outcomes

All patients recruited in the phase III of the GLORIA-AF registry underwent a 3-year follow-up, in which the incidence of major outcomes was recorded. For the purposes of this analysis, we evaluated the following outcomes, according to number of CKM domains, and CKM groups:

- All-cause mortality;
- Major adverse cardiovascular events (MACE, defined as the composite of cardiovascular death, stroke, and myocardial infarction);
- Thromboembolism (defined as a composite of stroke, TIA and other, non-central nervous system thromboembolism);
- Major Bleeding (defined as a life-threatening or fatal bleeding, symptomatic bleeding in a critical organ, or a bleeding associated with a haemoglobin reduction of ≥ 20 g/L or leading to ≥ 2 units of blood transfusion).

For this analysis, we considered the composite of all-cause death and MACE as our primary outcome, and evaluated the other outcomes as exploratory secondary outcomes.

### Statistical analysis

Continuous variables were reported as either mean ± standard deviation (SD) or median and interquartile range [IQR], and were compared with parametric or non-parametric tests, respectively. Frequencies (percentages) were used to represent categorical variables, which were compared with chi-square test.

The association of geographical region of recruitment (i.e., Europe, North America, Asia or Latin America) with odds of presenting with each CKM domain was analyzed using multiple-adjusted logistic regression models. We defined a base set of covariates for adjustments, which included the components of the CHA_2_DS_2_-VASc score (i.e., age class [< 65, 65–75, or ≥ 75 years], sex, arterial hypertension, diabetes mellitus, CHF, CAD, PAD, and history of stroke/TIA), BMI, CrCl, type of AF (either paroxysmal, persistent or permanent), history of previous bleeding, and hyperlipidemia; then, for each CKM domain, we fitted a logistic regression model adjusted for all those covariates, except those used to define that domain (i.e., for the regression on cardiovascular domain, we excluded from covariates CHF, CAD, PAD and history of stroke/TIA; for the kidney domain, we excluded CrCl; for the metabolic domain, we excluded diabetes, BMI and hyperlipidemia). Results were reported as Odds Ratio (OR) and 95% Confidence Intervals (CI).

The odds of receiving an oral anticoagulant (OAC), and NOACs vs. VKAs in patients prescribed an OAC, were analyzed using multiple-adjusted logistic regression models. Covariates included the components of the CHA_2_DS_2_-VASc score, geographical region of recruitment, BMI, CrCl, type of AF, history of previous bleeding, and hyperlipidemia. Results were reported as OR and 95%CI.

For the primary composite outcome of all-cause death and MACE, we assessed Kaplan–Meier curves according to numbers of CKM domains and CKM groups, and compared survival distributions using log-rank test; p values were adjusted with Benjamini–Hochberg method. For groups of CKM, we represented survival curves in cardiovascular, kidney, and metabolic panels, to improve visualization.

For all outcomes we reported incidence rate (IR) per 100 patients/year and 95%CI, according to number of CKM domains and CKM groups. We also performed multiple-adjusted Cox regressions to evaluate the hazard of the primary and secondary outcomes. Covariates included were components of the CHA_2_DS_2_-VASc score, geographical region of recruitment, BMI, CrCl, type of AF, history of previous bleeding, hyperlipidemia and use of OAC at baseline. Results were reported as Hazard Ratio (HR) and 95%CI.

We finally performed a sensitivity analysis, by applying more restrictive definitions to the kidney and metabolic domains (i.e., CrCl < 30 ml/min instead of < 60 ml/min for the kidney domain; BMI ≥ 30 kg/m^2^ instead of ≥ 25 kg/m^2^, along with the other criteria, for the metabolic domain), and we evaluated the associations with the primary outcome according to the recalculated number of CKM domains and CKM groups.

A two-sided *p* < 0.05 was considered statistically significant. All analyses were performed using R 4.3.1 (R Core Team 2020, Vienna, Austria).

## Results

Of 21,300 eligible patients enrolled in the GLORIA-AF Phase III registry, 16,070 (75.4%; age 70.1 ± 10.4 years, 45.2% females) who had complete information on variables used to define CKM domains and the primary composite outcome were included in this analysis. Baseline characteristics according to the numbers of CKM domains and CKM groups are reported in Tables 1 and S1 in supplementary materials. Cardiovascular, kidney and metabolic domains were found in 46.3%, 29.1% and 81.9% of patients, respectively. Overall, 983 (6.1%) patients had 0 CKM domains; 6818 (42.4%) had 1 domain, 6338 (39.4%) had 2 domains, and 1931 (12.0%) had all the 3 domains.

**Table 1:** Baseline Characteristics according to number of CKM Domains at baseline

Variables	0 CKM Domain (n = 983)	1 CKM Domain (n = 6818)	2 CKM Domains (n = 6338)	3 CKM Domains (n = 1931)	*P*
Age, mean (SD)	65.6 (10.2)	67.8 (10.1)	70.9 (10.1)	78.0 (7.2)	< 0.001
Female Sex, n (%)	482/983 (49.0)	3118/6818 (45.7)	2660/6338 (42.0)	996/1931 (51.6)	< 0.001
BMI, median [IQR]	23.1 [21.8–24.2]	28.2 [24.9–32.5]	28.0 [25.2–31.9]	26.5 [24.2–29.3]	< 0.001
Region, n (%)					< 0.001
North America	135/983 (13.7)	1781/6818 (26.1)	1668/6338 (26.3)	485/1931 (25.1)	
Europe	414/983 (42.1)	3345/6818 (49.1)	3070/6338 (48.4)	978/1931 (50.6)	
Asia	395/983 (40.2)	1347/6818 (19.8)	1222/6338 (19.3)	324/1931 (16.8)	
Latin America	39/983 (4.0)	345/6818 (5.1)	378/6338 (6.0)	144/1931 (7.5)	
AF Type, n (%)					< 0.001
Paroxysmal AF	654/983 (66.5)	4054/6818 (59.5)	3465/6338 (54.7)	1037/1931 (53.7)	
Persistent AF	294/983 (29.9)	2266/6818 (33.2)	2238/6338 (35.3)	662/1931 (34.3)	
Permanent AF	35/983 (3.6)	498/6818 (7.3)	635/6338 (10.0)	232/1931 (12.0)	
*Symptoms, n (%)*					
EHRA III-IV	339/983 (34.5)	2144/6818 (31.4)	2151/6338 (33.9)	685/1931 (35.5)	0.001
*Medical History, n (%)*					
Hypertension	516/982 (52.5)	4971/6809 (73.0)	4884/6334 (77.1)	1631/1931 (84.5)	< 0.001
Heart Failure	0/983 (0.0)	258/6818 (3.8)	2384/6338 (37.6)	920/1931 (47.6)	< 0.001
CAD	0/983 (0.0)	169/6818 (2.5)	2042/6338 (32.2)	897/1931 (46.5)	< 0.001
Diabetes Mellitus	0/983 (0.0)	1369/6818 (20.1)	1756/6338 (27.7)	681/1931 (35.3)	< 0.001
Hyperlipidemia	0/983 (0.0)	2264/6818 (33.2)	3033/6338 (47.9)	1230/1931 (63.7)	< 0.001
PAD	0/983 (0.0)	18/6818 (0.3)	311/6338 (4.9)	163/1931 (8.4)	< 0.001
Previous Stroke/TIA	0/983 (0.0)	196/6818 (2.9)	1557/6338 (24.6)	688/1931 (35.6)	< 0.001
Previous Bleeding	30/968 (3.1)	298/6753 (4.4)	414/6253 (6.6)	158/1900 (8.3)	< 0.001
Chronic Obstructive Pulmonary Disease	39/975 (4.0)	316/6790 (4.7)	467/6309 (7.4)	192/1918 (10.0)	< 0.001
Dementia	3/975 (0.3)	14/6797 (0.2)	45/6308 (0.7)	33/1919 (1.7)	< 0.001
History of Cancer	86/970 (8.9)	675/6762 (10.0)	627/6290 (10.0)	264/1916 (13.8)	< 0.001
Creatinine Clearance ≥ 60 ml/min	983/983 (100.0)	6081/6818 (89.2)	4326/6338 (68.3)	0/1931 (0.0)	< 0.001
*Risk scores*					
CHA_2_DS_2_-VASc, mean (SD)	1.9 (0.9)	2.5 (1.1)	3.7 (1.4)	4.9 (1.4)	< 0.001
HAS-BLED, mean (SD)	1.0 (0.8)	1.1 (0.8)	1.6 (0.9)	2.0 (0.9)	< 0.001

BMI, Body Mass Index; CAD, Coronary Artery Disease; EHRA, European Heart Rhythm Association; IQR, Interquartile Range; PAD, Peripheral Artery Disease; SD, Standard Deviation; TIA, Transient Ischemic Attack

Among groups of CKM, the metabolic only (5535, 34.4%) and the cardio-metabolic (4326, 26.9%) were the most prevalent. Patients with a higher number of CKM domains were older, more likely females, and with a high prevalence of most cardiovascular and non-cardiovascular comorbidities. They also showed higher mean CHA_2_DS_2_-VASc scores (Table 1). Similar results were observed when stratifying patients according to group of CKM (Table S1), with burden of comorbidities and risk factors that increased in the more complex groups.

### Geographical distribution of CKM

**Fig. 1:**
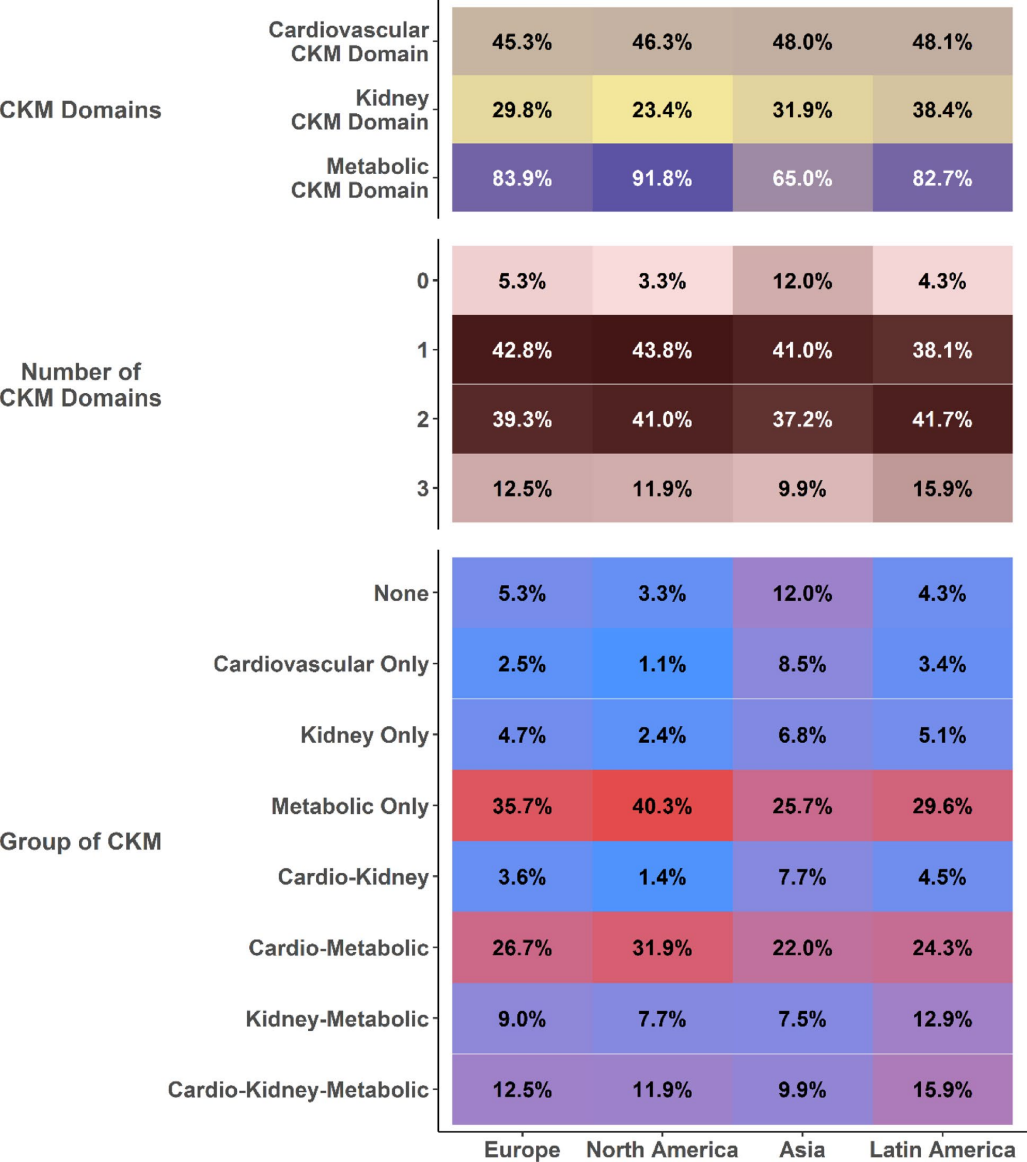
CKM domains, number of CKM domains and CKM groups according to geographical region of recruitment. CKM, Cardio-Kidney-Metabolic

Distributions of CKM domains, number and groups according to geographical regions are reported in Fig. 1. The prevalence of cardiovascular domain was similar across all regions. Conversely, kidney domain was more prevalent in Asia (31.9%) and Latin America (38.4%), while the metabolic domain was least represented in Asia (65.0%) and most prevalent in North America (91.8%). On multiple-adjusted regression analysis, compared to patients recruited in Europe, Asian patients showed higher odds of presenting with the cardiovascular and kidney domains (OR [95%CI]: 1.28 [1.17–1.40] and 1.13 [1.01–1.27], respectively), and lower odds of presenting with the metabolic domain (OR [95%CI]: 0.38 [0.34–0.42]). Patients recruited in Latin America showed higher odds of presenting with kidney domain (OR [95%CI]: 1.62 [1.36–1.94], while patients recruited in North America had higher odds of presenting with the metabolic domain (OR [95%CI]: 1.75 [1.53–2.01]) (Fig. S1).

Compared to other regions, patients recruited in Asia more commonly presented with 0 CKM domains (12.0% vs. 5.3% in Europe, 3.3% in North America, and 4.3% in Latin America); conversely, North American showed higher prevalence of the metabolic only and cardio-metabolic groups (40.3% vs. 35.7% and 31.9% vs. 26.7% in North America and Europe, respectively). The prevalence of all CKM domains combined was highest in patients recruited in Latin America (15.9%) (Fig. 1).

### Treatments according to number of CKM domains and CKM groups

**Fig. 2:**
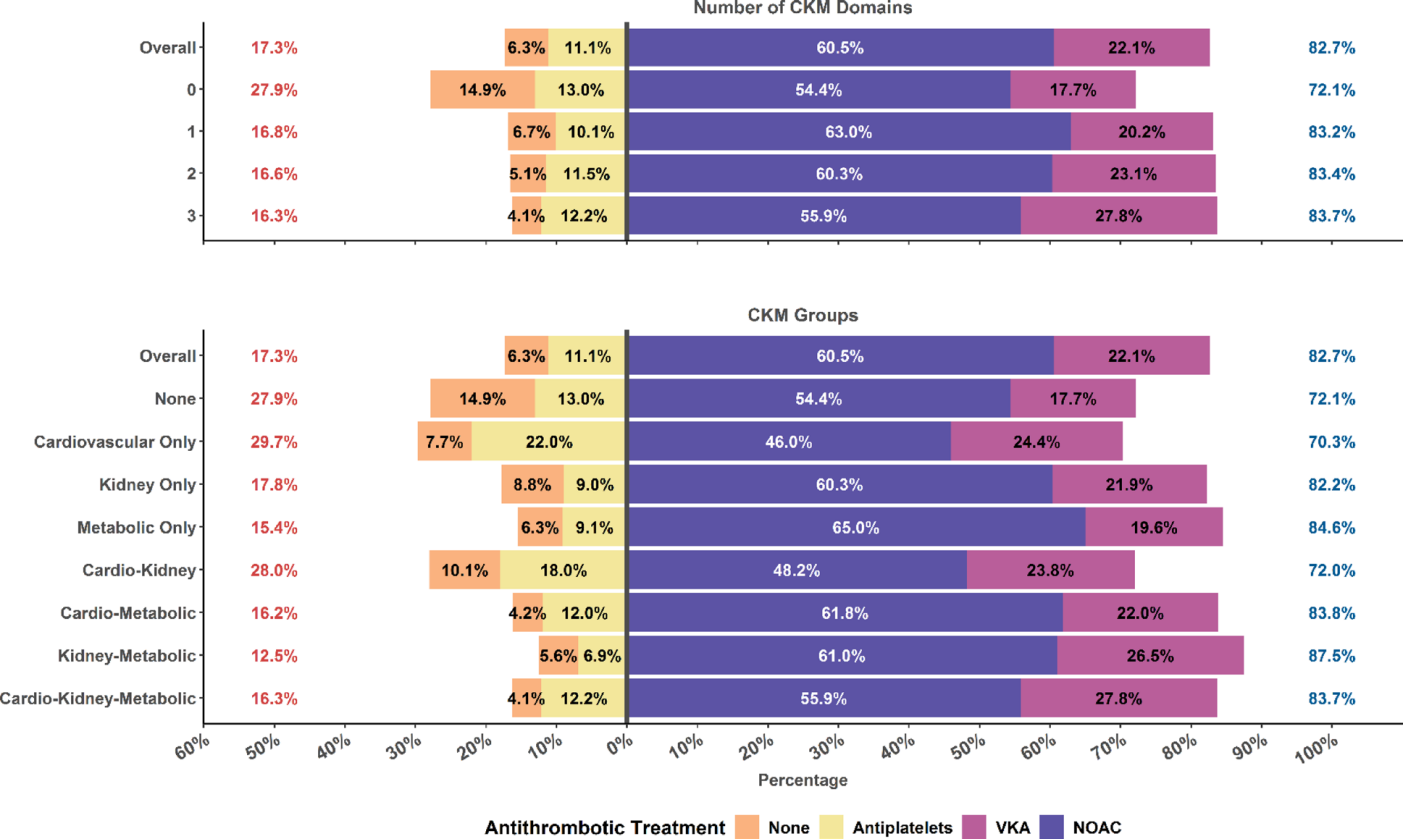
Use of antithrombotics according to number of CKM domains and CKM groups. NOAC, Non-vitamin K antagonist oral anticoagulant; VKA, Vitamin K Antagonist

Antithrombotic use is reported in Fig. 2. Compared to patients with 0 CKM domains, OAC were more used in patients with 1, 2 or 3 CKM domains (72.1% vs. 83.2%, 83.4% and 83.7%, respectively). Use of OAC was numerically higher in all groups characterized by the metabolic domain of CKM; conversely, the use of antiplatelet drugs was more commonly observed in the cardiovascular only and cardio-kidney groups (22.0% vs. 18.0%, respectively).

On multiple-adjusted logistic regression analysis, increasing number of CKM domains was associated with higher odds of receiving OAC, although differences were statistically significant only for 2 vs. 0 domains (OR [95%CI]: 1.40 [1.14–1.72]) and 3 vs. 0 domains (OR [95%CI]: 1.38 [1.07–1.78]) (Table 2). Compared to patients without any CKM domain, all groups were associated with higher odds of receiving OAC at baseline, except for the metabolic only and cardio-kidney groups (Table 2). Among patients who received an OAC, no statistically significant differences were observed for NOAC vs. VKA use according to numbers of CKM domains or groups of CKM (Table 2). Prevalences of the use of other drugs and treatments are reported in Tables S2 and S3 in Supplementary Materials.

**Table 2:** Multiple-adjusted Logistic Regression model on OAC use and NOAC vs. VKA use according to number and groups of CKM

	OAC vs. non-OAC use Odds Ratio [95%CI]	NOAC vs. VKA use Odds Ratio [95%CI]
*Number of CKM domains*		
0 CKM Domain	Ref	Ref
1 CKM Domain	1.18 [0.99–1.40]	0.98 [0.81–1.19]
2 CKM Domains	1.40 [1.14–1.72]	0.98 [0.79–1.22]
3 CKM Domains	1.38 [1.07–1.78]	0.91 [0.70–1.19]
*Groups of CKM*		
None	Ref	Ref
Cardiovascular Only	1.54 [1.15–2.05]	0.84 [0.62–1.15]
Kidney Only	1.41 [1.09–1.82]	0.95 [0.73–1.23]
Metabolic Only	1.11 [0.92–1.33]	1.05 [0.85–1.28]
Cardio-Kidney	1.19 [0.89–1.59]	0.94 [0.69–1.27]
Cardio-Metabolic	1.64 [1.28–2.10]	1.18 [0.92–1.52]
Kidney-Metabolic	1.35 [1.06–1.72]	0.85 [0.67–1.07]
Cardio-Kidney-Metabolic	1.53 [1.16–2.01]	0.97 [0.74–1.28]

CI, Confidence Interval; CKM, Cardio-Kidney-Metabolic; NOAC, Non-vitamin K antagonist oral anticoagulant; OAC, Oral Anticoagulant; VKA, vitamin K antagonist

### Clinical outcomes according to number of CKM domains and CKM groups

**Fig. 3:**
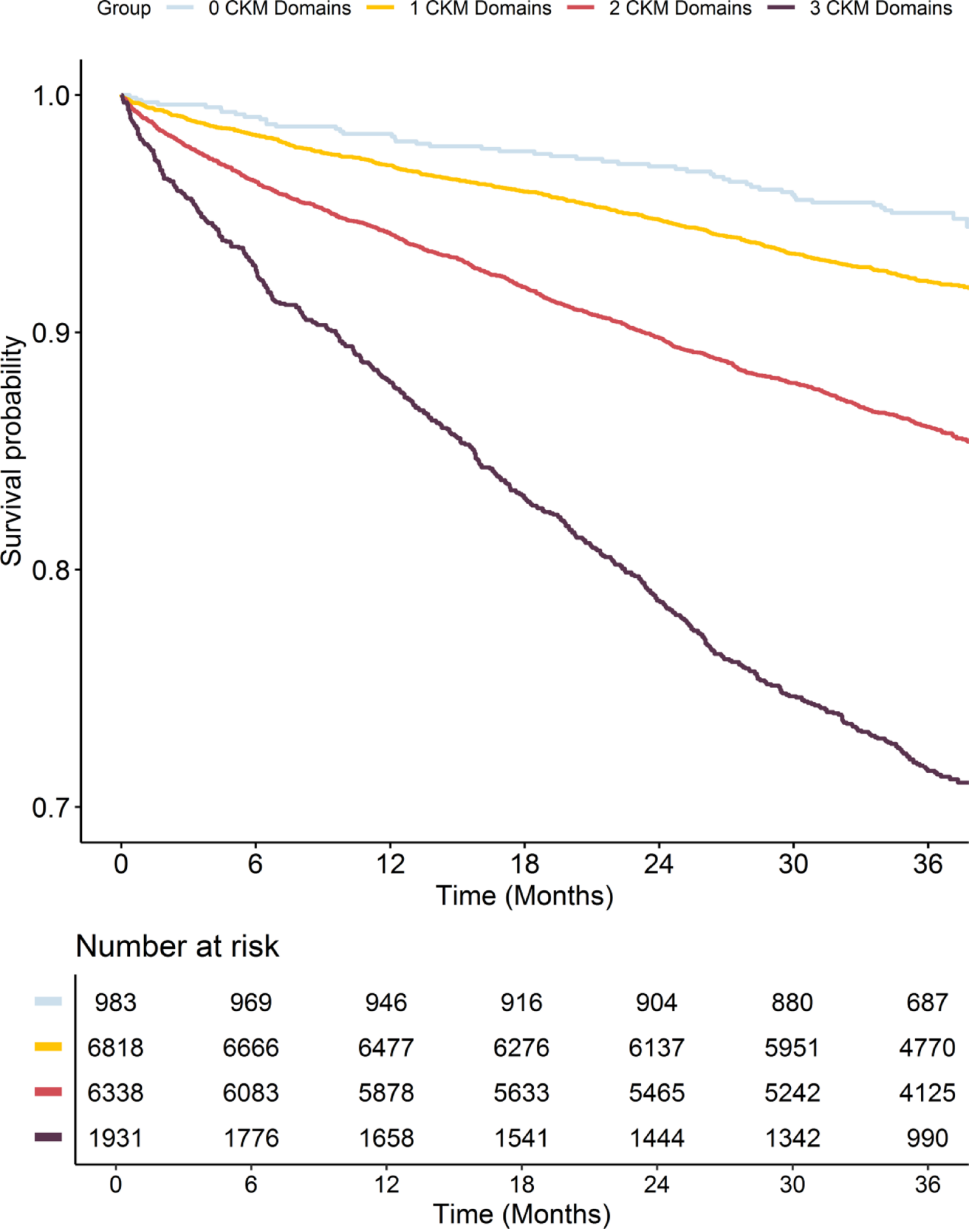
Kaplan–Meier curves for the primary composite outcome of all-cause death and MACE, according to number of CKM Domains. Log-Rank *p* = 0.002 for 0 vs. 1 Domain; < 0.001 for all other comparisons

**Fig. 4:**
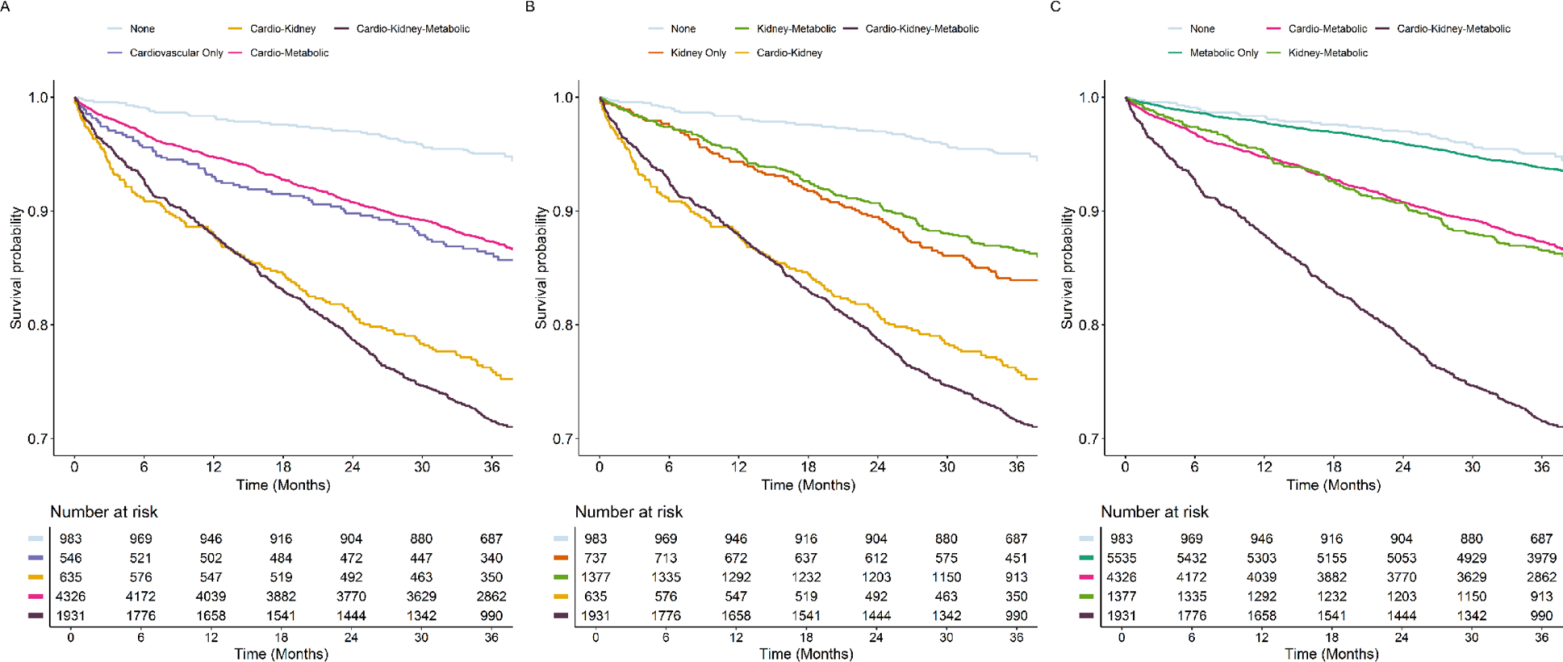
Kaplan–Meier curves for the primary composite outcome of all-cause death and MACE, according to groups of CKM. Panel A: Groups with Cardiovascular Domain; Panel B: Groups with Kidney Domain; Panel C: Groups with Metabolic Domain. Log-Rank p: 0.210 for none vs. metabolic only group; < 0.001 for all other groups vs. none

Over a median follow-up of 3.0 [2.9–3.1] years, the cumulative incidence of the primary composite outcome increased with the number of CKM domains, being highest in patients with 3 CKM domains (Fig. 3). Among groups of CKM, patients in the cardio-kidney and cardio-kidney-metabolic groups showed the highest incidence of the primary outcome (Fig. 4).

On multiple-adjusted Cox regression analyses, a higher number of CKM domains was associated with higher hazard of the primary composite outcome of all-cause death and MACE (HR [95%CI]: 1.37 [1.01–1.84], 1.40 [1.03–1.91] and 1.69 [1.20–2.37] for 0 vs. 1, 0 vs. 2 and 0 vs. 3 domains, respectively; Table 3). Similar results were observed for all-cause death, MACE and thromboembolism (although with statistical significance only for 3 vs. 0 domains for the latter two outcomes). No differences were observed for major bleeding.

**Table 3:** Multiple-adjusted Cox Regressions on the risk of Major Outcomes according to number of CKM domains and CKM groups

	All-Cause Death and MACE *IR [95%CI]* HR [95%CI]	All-Cause Death *IR [95%CI]* HR [95%CI]	MACE *IR [95%CI]* HR [95%CI]	Thromboembolism *IR [95%CI]* HR [95%CI]	Major Bleeding *IR [95%CI]* HR [95%CI]
*Number of CKM Domains*					
0 CKM Domain	*1.7 [1.3–2.3]* Ref	*1.1 [0.7–1.5]* Ref	*0.9 [0.6–1.4]* Ref	*0.7 [0.4–1.0]* Ref	*0.7 [0.4–1.0]* Ref
1 CKM Domain	*2.7 [2.5–3.0]* 1.37 [1.01–1.84]	*1.9 [1.7–2.1]* 1.50 [1.03–2.17]	*1.4 [1.3–1.6]* 1.34 [0.89–2.02]	*1.0 [0.9–1.1]* 1.39 [0.86–2.26]	*1.0 [0.8–1.1]* 1.13 [0.68–1.85]
2 CKM Domains	*5.1 [4.8–5.5]* 1.40 [1.03–1.91]	*3.8 [3.5–4.1]* 1.51 [1.03–2.21]	*2.7 [2.4–2.9]* 1.31 [0.85–2.01]	*1.6 [1.4–1.8]* 1.44 [0.86–2.40]	*1.5 [1.3–1.7]* 1.25 [0.74–2.11]
3 CKM Domains	*11.3 [10.4–12.3]* 1.69 [1.20–2.37]	*9.0 [8.2–9.8]* 1.75 [1.15–2.65]	*6.5 [5.8–7.3]* 1.81 [1.14–2.89]	*3.0 [2.5–3.5]* 1.81 [1.02–3.22]	*2.3 [1.9–2.8]* 1.14 [0.62–2.09]
*Groups of CKM*					
None	*1.7 [1.3–2.3]* Ref	*1.1 [0.7–1.5]* Ref	*0.9 [0.6–1.4]* Ref	*0.7 [0.4–1.0]* Ref	*0.7 [0.4–1.0]* Ref
Cardiovascular Only	*5.1 [4.0–6.4]* 1.57 [1.07–2.29]	*3.4 [2.5–4.5]* 1.54 [0.97–2.45]	*2.9 [2.1–4.0]* 1.63 [0.98–2.72]	*1.9 [1.3–2.7]* 1.89 [1.01–3.54]	*0.9 [0.5–1.6]* 1.30 [0.62–2.72]
Kidney Only	*5.7 [4.7–6.9]* 2.13 [1.51–3.01]	*4.6 [3.7–5.7]* 2.44 [1.60–3.70]	*2.6 [1.9–3.4]* 2.03 [1.25–3.29]	*1.8 [1.2–2.5]* 2.03 [1.15–3.59]	*1.1 [0.7–1.7]* 0.95 [0.50–1.81]
Metabolic Only	*2.1 [1.9–2.4]* 1.05 [0.77–1.44]	*1.4 [1.3–1.6]* 1.15 [0.78–1.69]	*1.2 [1.0–1.4]* 1.04 [0.68–1.59]	*0.8 [0.7–1.0]* 1.08 [0.65–1.80]	*1.0 [0.8–1.1]* 1.10 [0.66–1.85]
Cardio-Kidney	*9.7 [8.2–11.3]* 1.85 [1.30–2.64]	*7.8 [6.5–9.3]* 1.95 [1.27–3.00]	*5.0 [3.9–6.2]* 1.90 [1.17–3.09]	*2.7 [2.0–3.7]* 2.02 [1.11–3.69]	*2.1 [1.5–3.0]* 1.69 [0.89–3.21]
Cardio-Metabolic	*4.6 [4.2–5.0]* 1.12 [0.80–1.56]	*3.3 [3.0–3.6]* 1.18 [0.78–1.77]	*2.5 [2.2–2.8]* 1.04 [0.66–1.64]	*1.5 [1.3–1.7]* 1.36 [0.78–2.38]	*1.3 [1.1–1.6]* 1.18 [0.66–2.11]
Kidney-Metabolic	*4.8 [4.2–5.6]* 1.55 [1.10–2.17]	*3.7 [3.1–4.4]* 1.70 [1.12–2.56]	*2.3 [1.8–2.8]* 1.44 [0.90–2.29]	*1.4 [1.0–1.8]* 1.29 [0.74–2.27]	*1.8 [1.4–2.3]* 1.22 [0.69–2.14]
Cardio-Kidney-Metabolic	*11.3 [10.4–12.3]* 1.70 [1.21–2.40]	*9.0 [8.2–9.8]* 1.76 [1.16–2.68]	*6.5 [5.8–7.3]* 1.83 [1.14–2.93]	*3.0 [2.5–3.5]* 1.93 [1.08–3.45]	*2.3 [1.9–2.8]* 1.15 [0.62–2.14]

CI, Confidence Intervals; CKM, Cardio-Kidney-Metabolic; HR, Hazard Ratio; IR, Incidence Rate, MACE, Major Adverse Cardiovascular Events; Ref., Reference

Compared to patients without any CKM domain, all groups showed higher hazard of the primary composite outcome, except for the metabolic only and cardio-metabolic groups; highest magnitude of risk increase was observed for the kidney only group (HR [95%CI]: 2.13 [1.51–3.01]; Table 3). Similar results were observed for all-cause death. The kidney only, cardio-kidney, and cardio-kidney-metabolic groups also showed higher risk of MACE and thromboembolism. No differences were observed for major bleeding (Table 3).

### Sensitivity analyses

Results of the sensitivity analyses using more stringent criteria for the definition of CKM domains are reported in Table S4 in Supplementary Materials. Using alternate definitions, kidney and metabolic domains were found in 486 (3.0%) and 10,188 (63.4%) of patients, respectively. Broadly similar results for the association of number of CKM domains and CKM groups with risk of the primary outcome were obtained using the alternative definitions of kidney or metabolic domains (Table S4), although with some evidence of a diluted association for patients with less CKM domains, and greater association for groups of CKM which included kidney domain.

## Discussion

In this analysis from a contemporary prospective registry of patients with AF, our principal results are as follows: 1) components of the CKM syndrome were commonly found in patients with recently diagnosed AF, with significant geographical variation in the prevalence of the different CKM domains; 2) OAC use increased with the number of CKM domains, and was heterogeneously associated with groups of CKM, while no difference was observed for NOAC vs. VKA use according to CKM at adjusted regression analyses; 3) an increasing number of CKM domains was associated with higher incidence of major outcomes, including all-cause death, MACE and thromboembolism; the cardio-kidney and cardio-kidney-metabolic groups showed the highest cumulative incidence of all outcomes, while no differences were observed for major bleeding.

The concept of “cardio-kidney-metabolic” health was introduced to identify the connection among metabolic conditions (including obesity and diabetes), chronic kidney disease, and cardiovascular comorbidities, in view of the large impact that these conditions—and their interaction—have on the prognosis of the general population, and particularly on the risk of cardiovascular diseases [2]. Within this framework, AF is considered one of the terminal consequences of the CKM syndrome [2].

In this study, we show that conditions underpinning CKM domains are common in patients with established AF. We also show that prevalence of CKM domains increased with age, and is associated with relevant geographical differences. Particularly, metabolic CKM domain was more frequently observed in North America and less frequently observed in Asia, consistently with trends observed in the general population [15]. We also found that AF patients recruited in different geographical regions expressed different phenotypes of CKM. For example, North American patients more commonly had metabolic-driven groups of CKM, compared to European patients. These differences are important and suggest that CKM may express heterogeneously at a global level, likely due to geographical and ethnic differences in genetic and environmental factors, as well as the impact of social determinants of health, which increase the risk of CKM [2], and represent one of the emerging risk features in the pathogenesis and natural history of AF [16].

We also found that CKM influence thromboembolic risk prevention in patients with AF. Generally, the use of OAC increased with the burden of CKM domains, and shows variations across groups (i.e., phenotype) of CKM: indeed, adjusted regression analyses showed that CKM groups characterized by cardiovascular domain were generally more likely to receive OAC, while the contribution of the kidney domain was heterogenous across groups. Conversely, we did not observe significant differences in NOAC vs. VKA use across CKM groups. Taken together, these results show that specific combinations of comorbidities are associated with differences in the management of thromboembolic risk in patients with AF, as in previous studies [9, 17, 18]. Also, the accumulation of risk factors is an important driver of OAC prescription in contemporary patients with AF. In particular, the presence of cardiovascular risk factors seem to drive the use of OAC in our cohort, consistent with their prominent role in the CHA_2_DS_2_-VASc score, [5] which is used to guide decisions on OAC prescription.

Beyond treatments, we showed that an increasing burden of CKM domains was associated with a higher risk of adverse outcomes in patients with AF, with twofold higher incidence rates for each additional CKM domain accrued. These results were confirmed on adjusted Cox-regression analyses, which showed a dose–response relationship between the number of CKM domains and the risk of all-cause death and MACE. We additionally observed that groups characterized by the kidney domain had the highest relative increase in the risk of the primary outcome, while the metabolic domain appears to confer a lower increase in the risk of outcomes, particularly when found alone. On the other hand, we did not observe any statistical difference for the risk of major bleeding. Our sensitivity analyses applying more stringent criteria for kidney and metabolic domains confirm that incidence of adverse events was greater in patients with more advanced renal disease, and that risk increased throughout the decline of renal function.

These results have clinical implications, and show the complex interaction of the different CKM conditions in determining the prognosis of patients with AF. Previous studies have demonstrated the individual contribution of conditions underpinning CKM on the trajectories of patients with AF[19–22], and how the complexity of clinical phenotypes drives prognosis in these patients, particularly when both cardiovascular and non-cardiovascular risk factors are found [17, 23, 24]. Indeed, the contribution of an “unhealthy” metabolic status is associated with both an higher risk of AF incidence[25] and worse clinical outcomes in patients with AF, even across the spectrum of BMI [26]. Similarly, decreasing renal function is a known risk factor for all-cause death and overall worse prognosis in patients with AF [27, 28].

Our data expand on these findings, and suggest that the accumulation of CKM conditions has a synergistic detrimental effect on the prognosis of patients with AF, entailing a scenario of “clinical complexity” which bolster the risk of all-cause death, MACE and thromboembolism. Of note, we also found a numerically higher incidence of major bleeding in the more complex CKM group, and the lack of statistical significance should be interpreted in the context of the overall low incidence of major bleeding, potentially leading to reduced power to detect differences between subgroups.

Taken together, our results show that CKM syndrome is common in patients with AF, and represent a potentially actionable target for improving prognosis in these patients. Indeed, despite high rates of OAC use, AF patients with complex phenotypes and a higher burden of CKM still show a significantly higher risk of major outcomes, which requires additional strategies to improve their prognosis. In this context, screening and detection of CKM syndrome in patients with AF may allow for a more comprehensive approach to the management of CKM-associated complexity.

Current international recommendations for the management of AF advocate for a central role of the management of comorbidities and concurrent conditions [29–32], and such approach can be streamlined according to the evidence-based ‘Atrial fibrillation Better Care’ (ABC) pathway[28, 29, 32], or one of the other ABC-equivalent (but untested) acronym variants, such as ‘SOS’ or ‘CARE’ [29, 30, 33]. The ABC pathway has been shown to result in improved outcomes in patients with AF in two cluster randomized trials [34, 35], as well as in observational studies and meta-analysis [36–38]. As the ABC pathway has also been shown to benefit in specific subgroup of AF patients with non-cardiovascular comorbidities [39] and those deemed to be “clinically complex” [39, 40], such a holistic or integrated care approach is expected to represent a suitable strategy to improve outcomes also in AF patients with CKM complexity. Therefore, active screening for CKM domains may represent an opportunity to identify potentially actionable targets in patients with AF, recognizing also the geographical differences in the expression of CKM syndrome that we observed in our study. Finally, other pharmacological strategies, which has already proven effective in improving outcomes across the CKM spectrum [40, 41], may also represent promising candidates to improve outcomes in patients with AF and CKM.

### Strength and limitations

We used data from a global and large cohort of patients with AF, and to our knowledge this is one of the largest analyses on the impact of CKM domains in a prospective cohort of patients with established AF. Nonetheless, we acknowledge some limitations. First, our definitions of CKM domains were arbitrary, and based on the data available in our study; we did not have data on glucose or lipid levels, and other factors which could have been useful to better characterize some of the CKM domains [2]. Moreover, the GLORIA-AF Registry phase III enrolled patients with a recent diagnosis of AF over a limited time period (2014–2016); therefore caution should be used when translating our findings on other cohorts, and external validations are needed to confirm our results in different populations, particularly regarding the association with OAC, and NOAC vs. VKA use, considering that practice changes and increasing uptake of NOACs in more recent years may lead to different estimates in more contemporary practice. Additionally, we focused our analysis on patients who had complete data to define CKM and on the incidence of the primary outcome, and excluded those with missing data. As such, this could have introduced some bias in our analyses, and our results will need further validation in other cohorts. Despite the relatively large sample size of our analyses, we had limited power to detect differences in some subgroups, and therefore the lack of statistically significant differences (particularly regarding secondary outcomes) should be interpreted with caution. We also provided adjustments for the most relevant confounders on the relationship of CKM with OAC use and outcomes, but we cannot exclude the contribution of other unaccounted confounders. Also, as all patients enrolled in the GLORIA-AF patients had an established diagnosis of AF (which is one of the clinical manifestations of CKM syndrome), we were unable to evaluate the progression of CKM across different stages. Finally, our results on secondary outcomes were not adjusted for multiple comparisons, and should be therefore interpreted cautiously and be regarded as exploratory.

## Conclusions

In patients with AF, CKM domains are commonly found, and their prevalence is heterogeneous across geographical regions. CKM syndrome influence AF management and has detrimental prognostic effects, with an increasing risk of all-cause death and MACE as the burden of CKM domains increased.

## Supplementary Information

Below is the link to the electronic supplementary material.

Supplementary Material 1

Supplementary Material 2

## Data Availability

Data supporting this study by the data contributors Boehringer Ingelheim, and were made and are available through Vivli, Inc. Access was provided after a proposal was approved by an independent review committee identified for this purpose and after receipt of a signed data sharing agreement.
