# Supplementary material for: Cardio-kidney-metabolic complexity in patients with atrial fibrillation: an analysis from the prospective GLORIA-AF registry phase III
Source: Cardiovasc Diabetol. 2025 Oct 11;24:395. doi: 10.1186/s12933-025-02950-y (PMC12514827; doi:10.1186/s12933-025-02950-y)
Supplement: Supplementary file 1 — Supplementary Material 1 [file 12933_2025_2950_MOESM1_ESM.docx]

**Cardio-Kidney-Metabolic Complexity in patients with Atrial Fibrillation: an analysis from the prospective GLORIA-AF Registry Phase III**

Giulio Francesco Romiti, MD PhD^1,2^*, Davide Antonio Mei, MD^1,3^*, Bernadette Corica, MD^1,3^*, Marco Proietti, MD PhD^4,5^, Giuseppe Boriani, MD PhD^3^, Brian Olshansky, MD^6^, Tze-Fan Chao, MD^7,8,†^, Menno V Huisman, MD^9,§^,

Gregory Y. H. Lip, MD^1,10,11,†^**^,^**^§^; on behalf of the GLORIA-AF Investigators

**SUPPLEMENTARY MATERIALS**

**Table S1 – Baseline Characteristics according to groups of CKM**

| **Variables** | **None (n=983)** | **Cardiovascular Only (n=546)** | **Kidney Only (n=737)** | **Metabolic Only (n=5535)** | **Cardio-Kidney (n=635)** | **Cardio-Metabolic (n=4326)** | **Kidney-Metabolic (1377)** | **Cardio-Kidney-Metabolic (n=1931)** | **P** |
| --- | --- | --- | --- | --- | --- | --- | --- | --- | --- |
| Age, mean (SD) | 65.6 (10.2) | 65.8 (11.2) | 77.0 (7.1) | 66.8 (9.8) | 77.7 (7.6) | 68.0 (9.8) | 77.5 (7.0) | 78.0 (7.2) | <0.001 |
| Female Sex, n (%) | 482/983 (49.0) | 155/546 (28.4) | 442/737 (60.0) | 2521/5535 (45.5) | 326/635 (51.3) | 1412/4326 (32.6) | 922/1377 (67.0) | 996/1931 (51.6) | <0.001 |
| BMI, median [IQR] | 23.1 [21.8-24.2] | 23.2 [21.8-24.2] | 22.3 [20.6-23.5] | 29.6 [26.7-33.7] | 22.3 [20.7-23.7] | 29.4 [26.5-33.7] | 26.7 [24.7-29.0] | 26.5 [24.2-29.3] | <0.001 |
| **Region, n (%)** |  |  |  |  |  |  |  |  | <0.001 |
| North America | 135/983 (13.7) | 44/546 (8.1) | 99/737 (13.4) | 1638/5535 (29.6) | 57/635 (9.0) | 1298/4326 (30.0) | 313/1377 (22.7) | 485/1931 (25.1) |  |
| Europe | 414/983 (42.1) | 193/546 (35.3) | 367/737 (49.8) | 2785/5535 (50.3) | 283/635 (44.6) | 2085/4326 (48.2) | 702/1377 (51.0) | 978/1931 (50.6) |  |
| Asia | 395/983 (40.2) | 278/546 (50.9) | 225/737 (30.5) | 844/5535 (15.2) | 254/635 (40.0) | 723/4326 (16.7) | 245/1377 (17.8) | 324/1931 (16.8) |  |
| Latin America | 39/983 (4.0) | 31/546 (5.7) | 46/737 (6.2) | 268/5535 (4.8) | 41/635 (6.5) | 220/4326 (5.1) | 117/1377 (8.5) | 144/1931 (7.5) |  |
| **AF Type, n (%)** |  |  |  |  |  |  |  |  | <0.001 |
| Paroxysmal AF | 654/983 (66.5) | 296/546 (54.2) | 456/737 (61.9) | 3302/5535 (59.7) | 339/635 (53.4) | 2347/4326 (54.3) | 779/1377 (56.6) | 1037/1931 (53.7) |  |
| Persistent AF | 294/983 (29.9) | 209/546 (38.3) | 210/737 (28.5) | 1847/5535 (33.4) | 221/635 (34.8) | 1582/4326 (36.6) | 435/1377 (31.6) | 662/1931 (34.3) |  |
| Permanent AF | 35/983 (3.6) | 41/546 (7.5) | 71/737 (9.6) | 386/5535 (7.0) | 75/635 (11.8) | 397/4326 (9.2) | 163/1377 (11.8) | 232/1931 (12.0) |  |
| **Symptoms, n (%)** |  |  |  |  |  |  |  |  |  |
| EHRA III-IV | 339/983 (34.5) | 201/546 (36.8) | 220/737 (29.9) | 1723/5535 (31.1) | 220/635 (34.6) | 1522/4326 (35.2) | 409/1377 (29.7) | 685/1931 (35.5) | <0.001 |
| **Medical History, n (%)** |  |  |  |  |  |  |  |  |  |
| Hypertension | 516/982 (52.5) | 266/546 (48.7) | 459/737 (62.3) | 4246/5526 (76.8) | 420/635 (66.1) | 3335/4322 (77.2) | 1129/1377 (82.0) | 1631/1931 (84.5) | <0.001 |
| Heart Failure | 0/983 (0.0) | 258/546 (47.3) | 0/737 (0.0) | 0/5535 (0.0) | 340/635 (53.5) | 2044/4326 (47.2) | 0/1377 (0.0) | 920/1931 (47.6) | <0.001 |
| CAD | 0/983 (0.0) | 169/546 (31.0) | 0/737 (0.0) | 0/5535 (0.0) | 189/635 (29.8) | 1853/4326 (42.8) | 0/1377 (0.0) | 897/1931 (46.5) | <0.001 |
| Diabetes Mellitus | 0/983 (0.0) | 0/546 (0.0) | 0/737 (0.0) | 1369/5535 (24.7) | 0/635 (0.0) | 1340/4326 (31.0) | 416/1377 (30.2) | 681/1931 (35.3) | <0.001 |
| Hyperlipidemia | 0/983 (0.0) | 0/546 (0.0) | 0/737 (0.0) | 2264/5535 (40.9) | 0/635 (0.0) | 2353/4326 (54.4) | 680/1377 (49.4) | 1230/1931 (63.7) | <0.001 |
| PAD | 0/983 (0.0) | 18/546 (3.3) | 0/737 (0.0) | 0/5535 (0.0) | 32/635 (5.0) | 279/4326 (6.4) | 0/1377 (0.0) | 163/1931 (8.4) | <0.001 |
| Previous Stroke/TIA | 0/983 (0.0) | 196/546 (35.9) | 0/737 (0.0) | 0/5535 (0.0) | 223/635 (35.1) | 1334/4326 (30.8) | 0/1377 (0.0) | 688/1931 (35.6) | <0.001 |
| Previous Bleeding | 30/968 (3.1) | 24/544 (4.4) | 44/730 (6.0) | 230/5479 (4.2) | 43/629 (6.8) | 294/4265 (6.9) | 77/1359 (5.7) | 158/1900 (8.3) | <0.001 |
| Chronic Obstructive Pulmonary Disease | 39/975 (4.0) | 28/546 (5.1) | 41/733 (5.6) | 247/5511 (4.5) | 50/629 (7.9) | 351/4306 (8.2) | 66/1374 (4.8) | 192/1918 (10.0) | <0.001 |
| Dementia | 3/975 (0.3) | 2/546 (0.4) | 4/736 (0.5) | 8/5515 (0.1) | 11/631 (1.7) | 16/4301 (0.4) | 18/1376 (1.3) | 33/1919 (1.7) | <0.001 |
| History of Cancer | 86/970 (8.9) | 32/542 (5.9) | 101/728 (13.9) | 542/5492 (9.9) | 86/629 (13.7) | 386/4292 (9.0) | 155/1369 (11.3) | 264/1916 (13.8) | <0.001 |
| Creatinine Clearance ≥60 ml/min | 983/983 (100.0) | 546/546 (100.0) | 0/737 (0.0) | 5535/5535 (100.0) | 0/635 (0.0) | 4326/4326 (100.0) | 0/1377 (0.0) | 0/1931 (0.0) | <0.001 |
| **Risk Scores** |  |  |  |  |  |  |  |  |  |
| CHA_2_DS_2_-VASc, mean (SD) | 1.9 (0.9) | 2.9 (1.4) | 2.9 (0.9) | 2.4 (1.1) | 4.2 (1.3) | 3.7 (1.5) | 3.5 (1.0) | 4.9 (1.4) | <0.001 |
| HAS-BLED, mean (SD) | 1.0 (0.8) | 1.5 (1.0) | 1.3 (0.6) | 1.1 (0.8) | 1.8 (1.0) | 1.6 (1.0) | 1.4 (0.7) | 2.0 (0.9) | <0.001 |

**Legend:** BMI= Body Mass Index; CAD= Coronary Artery Disease; EHRA= European Heart Rhythm Association; IQR= Interquartile Range; PAD= Peripheral Artery Disease; SD= Standard Deviation; TIA= Transient Ischemic Attack.

**Table S2 – Treatments according to number of CKM domains**

| **Variables** | **0 CKM Domain  (n=983)** | **1 CKM Domain (n=6818)** | **2 CKM Domains (n=6338)** | **3 CKM Domains (n=1931)** | **P** |
| --- | --- | --- | --- | --- | --- |
| **Antithrombotic Treatment, n (%)** |  |  |  |  | <0.001 |
| None | 146/983 (14.9) | 457/6816 (6.7) | 323/6334 (5.1) | 80/1930 (4.1) |  |
| Antiplatelets | 128/983 (13.0) | 691/6816 (10.1) | 726/6334 (11.5) | 235/1930 (12.2) |  |
| NOAC | 535/983 (54.4) | 4291/6816 (63.0) | 3819/6334 (60.3) | 1078/1930 (55.9) |  |
| VKA | 174/983 (17.7) | 1377/6816 (20.2) | 1466/6334 (23.1) | 537/1930 (27.8) |  |
| **Other Treatments, n (%)** |  |  |  |  |  |
| Beta-Blockers | 500/983 (50.9) | 4129/6818 (60.6) | 4274/6338 (67.4) | 1355/1931 (70.2) | <0.001 |
| Verapamil/Diltiazem | 66/983 (6.7) | 612/6818 (9.0) | 444/6338 (7.0) | 113/1931 (5.9) | <0.001 |
| Amiodarone | 117/983 (11.9) | 846/6818 (12.4) | 983/6338 (15.5) | 328/1931 (17.0) | <0.001 |
| Dronedarone | 11/983 (1.1) | 76/6818 (1.1) | 48/6338 (0.8) | 7/1931 (0.4) | 0.008 |
| Digoxin | 40/983 (4.1) | 380/6818 (5.6) | 656/6338 (10.4) | 182/1931 (9.4) | <0.001 |
| Propafenone | 56/983 (5.7) | 267/6818 (3.9) | 112/6338 (1.8) | 15/1931 (0.8) | <0.001 |
| Flecainide | 66/983 (6.7) | 371/6818 (5.4) | 112/6338 (1.8) | 25/1931 (1.3) | <0.001 |
| Other Antiarrhythmics | 76/983 (7.7) | 344/6818 (5.0) | 412/6338 (6.5) | 147/1931 (7.6) | <0.001 |
| AF Ablation/Cardioversion | 246/978 (25.2) | 1628/6796 (24.0) | 1375/6311 (21.8) | 336/1925 (17.5) | <0.001 |
| ACE inhibitors/ARB | 287/983 (29.2) | 3380/6818 (49.6) | 3830/6338 (60.4) | 1139/1931 (59.0) | <0.001 |
| Diuretics | 107/983 (10.9) | 2018/6818 (29.6) | 2885/6338 (45.5) | 1058/1931 (54.8) | <0.001 |
| Statins | 126/983 (12.8) | 2379/6818 (34.9) | 3629/6338 (57.3) | 1307/1931 (67.7) | <0.001 |
| Insulin | 0/983 (0.0) | 190/6818 (2.8) | 363/6338 (5.7) | 177/1931 (9.2) | <0.001 |
| Oral Hypoglycemic Agents | 2/983 (0.2) | 940/6818 (13.8) | 1137/6338 (17.9) | 372/1931 (19.3) | <0.001 |

Legend: ACE= Angiotensin Converting Enzyme; AF= Atrial Fibrillation; ARB= Angiotensin Receptor Blockers; NOAC= non-vitamin K antagonist oral anticoagulant; VKA= vitamin K antagonist

**Table S3 – Treatments according to groups of CKM**

| **Variables** | **None (n=983)** | **Cardiovascular Only (n=546)** | **Kidney Only (n=737)** | **Metabolic Only (n=5535)** | **Cardio-Kidney (n=635)** | **Cardio-Metabolic (n=4326)** | **Kidney-Metabolic (1377)** | **Cardio-Kidney-Metabolic (n=1931)** | **P** |
| --- | --- | --- | --- | --- | --- | --- | --- | --- | --- |
| **Antithrombotic Treatment, n (%)** |  |  |  |  |  |  |  |  | <0.001 |
| None | 146/983 (14.9) | 42/546 (7.7) | 65/736 (8.8) | 350/5534 (6.3) | 64/635 (10.1) | 182/4322 (4.2) | 77/1377 (5.6) | 80/1930 (4.1) |  |
| Antiplatelets | 128/983 (13.0) | 120/546 (22.0) | 66/736 (9.0) | 505/5534 (9.1) | 114/635 (18.0) | 517/4322 (12.0) | 95/1377 (6.9) | 235/1930 (12.2) |  |
| NOAC | 535/983 (54.4) | 251/546 (46.0) | 444/736 (60.3) | 3596/5534 (65.0) | 306/635 (48.2) | 2673/4322 (61.8) | 840/1377 (61.0) | 1078/1930 (55.9) |  |
| VKA | 174/983 (17.7) | 133/546 (24.4) | 161/736 (21.9) | 1083/5534 (19.6) | 151/635 (23.8) | 950/4322 (22.0) | 365/1377 (26.5) | 537/1930 (27.8) |  |
| **Other Treatments, n (%)** |  |  |  |  |  |  |  |  |  |
| Beta-Blockers | 500/983 (50.9) | 315/546 (57.7) | 369/737 (50.1) | 3445/5535 (62.2) | 378/635 (59.5) | 3069/4326 (70.9) | 827/1377 (60.1) | 1355/1931 (70.2) | <0.001 |
| Verapamil/Diltiazem | 66/983 (6.7) | 17/546 (3.1) | 57/737 (7.7) | 538/5535 (9.7) | 37/635 (5.8) | 292/4326 (6.7) | 115/1377 (8.4) | 113/1931 (5.9) | <0.001 |
| Amiodarone | 117/983 (11.9) | 85/546 (15.6) | 112/737 (15.2) | 649/5535 (11.7) | 93/635 (14.6) | 688/4326 (15.9) | 202/1377 (14.7) | 328/1931 (17.0) | <0.001 |
| Dronedarone | 11/983 (1.1) | 0/546 (0.0) | 6/737 (0.8) | 70/5535 (1.3) | 1/635 (0.2) | 35/4326 (0.8) | 12/1377 (0.9) | 7/1931 (0.4) | 0.001 |
| Digoxin | 40/983 (4.1) | 72/546 (13.2) | 47/737 (6.4) | 261/5535 (4.7) | 99/635 (15.6) | 478/4326 (11.0) | 79/1377 (5.7) | 182/1931 (9.4) | <0.001 |
| Propafenone | 56/983 (5.7) | 15/546 (2.7) | 22/737 (3.0) | 230/5535 (4.2) | 9/635 (1.4) | 56/4326 (1.3) | 47/1377 (3.4) | 15/1931 (0.8) | <0.001 |
| Flecainide | 66/983 (6.7) | 13/546 (2.4) | 28/737 (3.8) | 330/5535 (6.0) | 5/635 (0.8) | 71/4326 (1.6) | 36/1377 (2.6) | 25/1931 (1.3) | <0.001 |
| Other Antiarrhythmics | 76/983 (7.7) | 44/546 (8.1) | 39/737 (5.3) | 261/5535 (4.7) | 52/635 (8.2) | 309/4326 (7.1) | 51/1377 (3.7) | 147/1931 (7.6) | <0.001 |
| AF Ablation/Cardioversion | 246/978 (25.2) | 102/544 (18.8) | 131/734 (17.8) | 1395/5518 (25.3) | 108/635 (17.0) | 1033/4307 (24.0) | 234/1369 (17.1) | 336/1925 (17.5) | <0.001 |
| ACE inhibitors/ARB | 287/983 (29.2) | 253/546 (46.3) | 257/737 (34.9) | 2870/5535 (51.9) | 310/635 (48.8) | 2767/4326 (64.0) | 753/1377 (54.7) | 1139/1931 (59.0) | <0.001 |
| Diuretics | 107/983 (10.9) | 177/546 (32.4) | 187/737 (25.4) | 1654/5535 (29.9) | 290/635 (45.7) | 2058/4326 (47.6) | 537/1377 (39.0) | 1058/1931 (54.8) | <0.001 |
| Statins | 126/983 (12.8) | 213/546 (39.0) | 98/737 (13.3) | 2068/5535 (37.4) | 249/635 (39.2) | 2775/4326 (64.1) | 605/1377 (43.9) | 1307/1931 (67.7) | <0.001 |
| Insulin | 0/983 (0.0) | 0/546 (0.0) | 0/737 (0.0) | 190/5535 (3.4) | 1/635 (0.2) | 289/4326 (6.7) | 73/1377 (5.3) | 177/1931 (9.2) | <0.001 |
| Oral Hypoglycemic Agents | 2/983 (0.2) | 2/546 (0.4) | 1/737 (0.1) | 937/5535 (16.9) | 2/635 (0.3) | 893/4326 (20.6) | 242/1377 (17.6) | 372/1931 (19.3) | <0.001 |

Legend: ACE= Angiotensin Converting Enzyme; AF= Atrial Fibrillation; ARB= Angiotensin Receptor Blockers; NOAC= non-vitamin K antagonist oral anticoagulant; VKA= vitamin K antagonist

**Table S4 – Multiple-adjusted Cox Regressions on the risk of primary outcome according to number of CKM domains and CKM groups, using alternate definitions for CKM Domain**

|  | **Number of CKM Domains** | | | | **Groups of CKM Domains** | | | | | | | |
| --- | --- | --- | --- | --- | --- | --- | --- | --- | --- | --- | --- | --- |
|  | **0** | **1** | **2** | **3** | **None** | **CV only** | **Kidney Only** | **Metabolic Only** | **Cardio-Kidney** | **Cardio-Metabolic** | **Kidney-Metabolic** | **Cardio-Kidney-Metabolic** |
| **Alternate definition for Kidney Domain** | | | | | | | | | | | | |
| N | 1650 | 7983 | 6201 | 236 | 1650 | 1119 | 70 | 6794 | 62 | 6021 | 118 | 236 |
| *IR [95%CI]* | 3.0 [2.5-3.5] | 3.2 [2.9-3.4] | 6.2 [5.9-6.6] | *22.4 [18.4-27.1]* | *3.0 [2.5-3.5]* | *6.7 [5.8-7.7]* | *14.6 [9.3-21.9]* | *2.5 [2.3-2.8]* | *25.9 [17.5-37.0]* | *6.5 [5.7-6.4]* | *10.0 [6.8-14.3]* | *22.4 [18.4-27.1]* |
| HR [95%CI] | Ref. | 0.99 [0.81-1.20] | 1.03 [0.82-1.30] | 1.77 [1.30-2.40] | Ref. | 1.10 [0.86-1.41] | 2.97 [1.90-4.66] | 0.83 [0.68-1.03] | 2.67 [1.76-4.05] | 0.91 [0.72-1.15] | 1.79 [1.18-2.72] | 1.78 [1.30-2.43] |
| **Alternate definition for Metabolic Domain** | | | | | | | | | | | | |
| N | 2397 | 6620 | 5473 | 1580 | 2397 | 1356 | 1143 | 4121 | 986 | 3516 | 971 | 1580 |
| *IR [95%CI]* | 1.8 [1.5-2.1] | 3.3 [3.0-3.5] | 5.6 [5.2-6.0] | *11.5 [10.4-12.6[]* | *1.8 [1.5-2.1]* | *4.7 [4.0-5.4]* | *5.4 [4.6-6.2]* | *2.3 [2.0-2.5]* | *10.0 [8.8-11.3]* | *4.6 [4.2-5.1]* | *4.9 [4.1-5.8]* | *11.5 [10.4-12.6]* |
| HR [95%CI] | Ref. | 1.52 [1.24-1.86] | 1.55 [1.23-1.95] | 1.85 [1.40-2.44] | Ref. | 1.47 [1.14-1.91] | 2.02 [1.58-2.60] | 1.16 [0.91-1.47] | 1.97 [1.51-2.56] | 1.13 [0.87-1.47] | 1.59 [1.19-2.12] | 1.71 [1.29-2.27] |
|  |  |  |  |  |  |  |  |  |  |  |  |  |

**Figure S1 – Multiple-adjusted regression on the association of CKM domains with geographical regions**

**
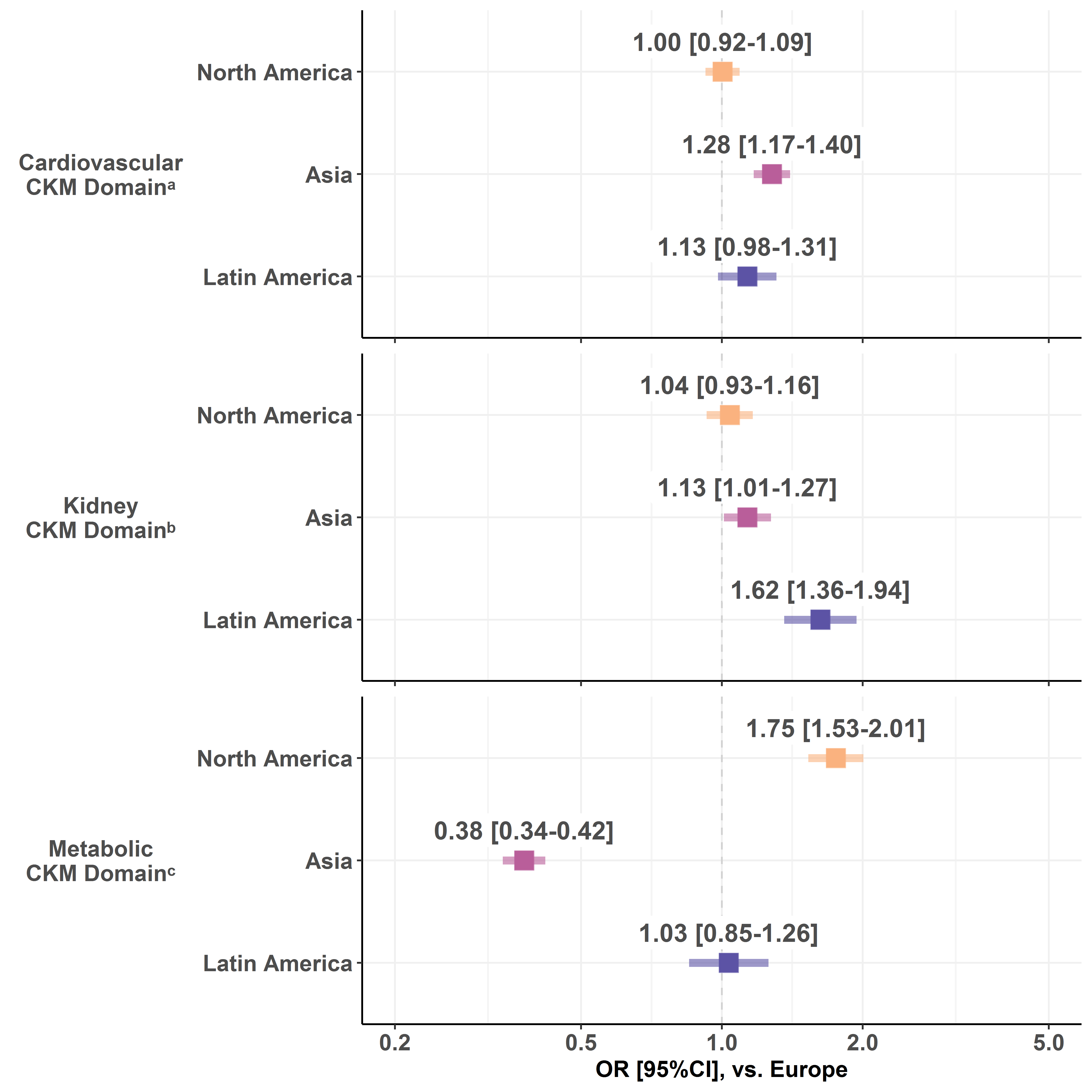
**

Legend: CKM= Cardio-Kidney-Metabolic. ^a^=adjusted for age class, sex, hypertension, diabetes, body mass index, creatinine clearance, hyperlipidemia, history of previous bleeding and type of AF; ^b^=adjusted for age class, sex, hypertension, diabetes, congestive heart failure, coronary artery disease, peripheral artery disease, history of stroke/TIA, body mass index, hyperlipidemia, history of previous bleeding, and type of AF; ^c^=adjusted for age class, sex, hypertension, congestive heart failure, coronary artery disease, peripheral artery disease, history of stroke/TIA, creatinine clearance, history of previous bleeding, and type of A
